# Prediction of Deleterious Non-Synonymous SNPs Based on Protein Interaction Network and Hybrid Properties

**DOI:** 10.1371/journal.pone.0011900

**Published:** 2010-07-30

**Authors:** Tao Huang, Ping Wang, Zhi-Qiang Ye, Heng Xu, Zhisong He, Kai-Yan Feng, LeLe Hu, WeiRen Cui, Kai Wang, Xiao Dong, Lu Xie, Xiangyin Kong, Yu-Dong Cai, Yixue Li

**Affiliations:** 1 Key Laboratory of Systems Biology, Shanghai Institutes for Biological Sciences, Chinese Academy of Sciences, Shanghai, People's Republic of China; 2 Shanghai Center for Bioinformation Technology, Shanghai, People's Republic of China; 3 Institute of Health Sciences, Shanghai Institutes for Biological Sciences, Chinese Academy of Sciences and Shanghai Jiao Tong University School of Medicine, Shanghai, People's Republic of China; 4 State Key Laboratory of Medical Genomics, Ruijin Hospital, Shanghai Jiaotong University, Shanghai, People's Republic of China; 5 Institute of Systems Biology, Shanghai University, Shanghai, People's Republic of China; 6 Centre for Computational Systems Biology, Fudan University, Shanghai, People's Republic of China; 7 CAS-MPG Partner Institute for Computational Biology, Shanghai Institutes for Biological Sciences, Chinese Academy of Sciences, Shanghai, People's Republic of China; Aarhus University, Denmark

## Abstract

Non-synonymous SNPs (nsSNPs), also known as Single Amino acid Polymorphisms (SAPs) account for the majority of human inherited diseases. It is important to distinguish the deleterious SAPs from neutral ones. Most traditional computational methods to classify SAPs are based on sequential or structural features. However, these features cannot fully explain the association between a SAP and the observed pathophysiological phenotype. We believe the better rationale for deleterious SAP prediction should be: If a SAP lies in the protein with important functions and it can change the protein sequence and structure severely, it is more likely related to disease. So we established a method to predict deleterious SAPs based on both protein interaction network and traditional hybrid properties. Each SAP is represented by 472 features that include sequential features, structural features and network features. Maximum Relevance Minimum Redundancy (mRMR) method and Incremental Feature Selection (IFS) were applied to obtain the optimal feature set and the prediction model was Nearest Neighbor Algorithm (NNA). In jackknife cross-validation, 83.27% of SAPs were correctly predicted when the optimized 263 features were used. The optimized predictor with 263 features was also tested in an independent dataset and the accuracy was still 80.00%. In contrast, SIFT, a widely used predictor of deleterious SAPs based on sequential features, has a prediction accuracy of 71.05% on the same dataset. In our study, network features were found to be most important for accurate prediction and can significantly improve the prediction performance. Our results suggest that the protein interaction context could provide important clues to help better illustrate SAP's functional association. This research will facilitate the post genome-wide association studies.

## Introduction

Millions of single nucleotide polymorphisms (SNPs) have been collected in the public database, dbSNP [1], and it is estimated that ∼90% of human sequence variants are SNPs [2]. Among them, non-synonymous SNPs (nsSNPs), also known as single amino acid polymorphisms (SAPs), that lead to a single amino acid change in the protein product are most relevant to human inherited diseases [3]. Two databases, the Online Mendelian Inheritance in Man (OMIM) [4] and the Human gene mutation database (HGMD) [3], contain records of disease-causing variants and suggest that the majority of the disease-causing variants are non-synonymous changes [5]. It is estimated that there are 67,000–200,000 nsSNPs in the human population [5]. Some of these nsSNPs are disease-associated, while others are functionally neutral. It is important to discriminate disease-associated nsSNPs from neutral ones for the investigation of genetic diseases.

Empirical rule-based [6], [7], [8], probabilistic models [9] and machine learning approaches [10], [11], [12], [13], [14], [15], [16], [17] were used to classify the nsSNPs. These studies made use of a variety of potential features to distinguish deleterious nsSNPs from neutral ones – mainly features derived from protein sequences [11], [12], [13] or from both protein structural and sequential information [10], [14], [15], [16], [17]. However, only a limited number of proteins have known three-dimensional structures, while the vast majority does not have their structural information available [5]. Among the above mentioned papers that mainly used the sequence information, some did not consider the sequence microenvironment [13] and some lacked a feature selection procedure [16].

The major limitation of traditional methods that are based on structural or sequential features is that they only focus on the local variation of the protein itself. Although the prediction accuracy may be high, it is hard to believe that the change of only one SAP protein could determine or cause a pathophysiological phenotype. More and more studies have shown that diseases can be caused by perturbed cellular networks [18], [19]. Including network features, therefore, should improve the prediction of deleterious SAPs.

In this paper, a new classification method was established by combining new network features and traditional sequential features of the amino acid microenvironment surrounding the SAP and using a carefully designed feature selection procedure. Each SAP was coded by 472 features, which were derived from the transformed scores of the amino acid index, position-specific scoring matrices, the structural features, betweenness and the KEGG enrichment scores of the protein neighbors in STRING [20] network. Next, feature selection and analysis methods, including the Maximum Relevance Minimum Redundancy method (mRMR) [21] and Incremental Feature Selection (IFS) [22] were used to obtain the optimal features to be used for the prediction of deleterious nsSNPs versus neutral ones. The prediction model was built using well-known Nearest Neighbor Algorithm (NNA) [23]. As a result, the optimal 263-feature set were selected, achieving a correct prediction rate of 83.27% when evaluated by Jackknife cross-validation test. The optimized prediction model with 263 features was also tested on an independent dataset, and the accuracy was still 80.00%. Network features were found to be most important for accurate prediction.

## Materials and Methods

### Dataset

Care et al. [24] evaluated several common SAP (single amino acid polymorphism) datasets and concluded that the Swiss-Prot dataset is the best training data for the prediction of SAPs. In this study, SAP data from Swiss-Prot Protein Knowledgebase (http://www.uniprot.org/docs/humsavar, release 57.4 of 16-Jun-2009 and release 57.13 of 19-Jan-2010) were acquired for the prediction and analysis of SAPs. Human polymorphisms and disease mutations in release 57.4 were used for Jackknife cross-validation. The SAPs added in release 57.13 after release 57.4 were used as an independent test dataset. Each SAP in the Swiss-Prot is annotated with a label of either ‘disease’ (SAP with disease association), ‘polymorphism’ (SAP with no known disease association) or ‘unclassified’ (SAP which has too little information to be classified). We excluded ‘unclassified’ SAPs and SAPs without the required features for our method. The final, filtered dataset was composed of 20,706 polymorphism SAPs and 16,304 disease SAPs. The independent test dataset was composed of 1,905 polymorphism SAPs and 766 disease SAPs.

### Feature Construction

#### The features of the network

In a network, some nodes occupy important positions; others must rely on those nodes to exchange information. Such a network property of a node can be studied using Freeman's betweenness measure [25]. For a graph 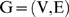, the betweenness of node 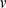 is defined as:
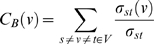
(1)where 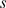 and 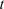 are all the other nodes in the network, 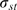 is the number of shortest paths between node 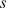 and node 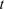 and 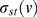 is the number of those paths that go through node 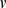.

Betweenness is used to measure information that flows through networks. High betweenness means that there are multiple paths between nodes, and low betweenness means there are few paths. In a biological network, betweenness measures the ways in which signals can pass through the interaction network. The R package tnet (http://opsahl.co.uk/tnet) was used to calculate the betweenness of each protein in the weighted network derived from STRING v8.2 [20].

The most simple and direct method to predict one protein's function is to consider the known functions of proteins found in its immediate neighborhood [26]. The function of neighbors is an important feature for the environment of this protein. The enrichment score of one protein's neighbors on a STRING network was defined as the −log_10_ of the p-value generated by the hypergeometric test. The larger the enrichment score of one KEGG pathway, the more overrepresented this pathway is. There were 220 KEGG enrichment score features. Betweenness and the KEGG enrichment scores were network level features.

#### The features of the PSSM conservation score

Evolutionary conservation is one of the most important concepts in biology. If an amino acid in a particular position of a particular protein is conserved, it indicates that this amino acid may be located in an important or functional region of the protein and that its mutation may cause a significant change of the protein's structure and function.

Position Specific Iterative BLAST (PSI BLAST) can measure the residue conservation at a given location. It uses a 20-dimensional vector to represent the probabilities of conservation against mutations to 20 different amino acids. Position Specific Scoring Matrix (PSSM) [27] is a matrix of such vectors which represent all residues in a given sequence. If a residue is conserved in PSI BLAST, it is likely to be important for biological function.

In this study, we used the PSSM conservation score to quantify the conservation status of each amino acid in the protein sequence. Target sequences were scanned against the reference data sets UniRef100 Release 15.9 to generate the position specific scoring matrices (PSSMs) using Position Specific Iterative BLAST (PSI BLAST) program Release 2.2.12 [28].

#### The features of the disorder score

Disordered regions in proteins lack fixed three-dimensional structures under physiological conditions, but they play important roles in regulation, signaling and control. These activities are achieved by high-specificity, low-affinity interactions and the binding of multiple proteins [29]. Amino acid substitutions occurring in these regions would, presumably, disturb their normal functions and thereby demonstrate a “disease” phenotype. Previous investigations have proven that disordered regions can contribute to the prediction of SAP disease association [16].

In this study, we used the disorder score, calculated by VSL2 [30], to quantify the disorder status of each amino acid in the protein sequence. VSL2 can predict disordered regions of any length, and it can accurately identify short disordered regions. The disorder scores of the surrounding amino acids of the SAP site formed the features of disorder.

#### The features of AAFactors

AAIndex (http://www.genome.ad.jp/aaindex/) is a database of numerical indices, representing various physicochemical and biochemical properties of amino acids or pairs of amino acids. Atchley et al. [31] did factor analysis on AAIndex to produce a small set of highly interpretable numeric patterns of amino acid variability. These high-dimensional attributes of amino acids were summarized and transformed to five multidimensional patterns of attribute covariation that reflected polarity, secondary structure, molecular volume, codon diversity, and electrostatic charge. These five transformed scores (we called “amino acid factors” or “AAFactors”) were used to encode each amino acid in our research.

#### Other structural features

Twelve features in Ye's study [16] were also included in our feature space. These features were described as follows:

HLA family. HLA is a group of genes with diverse functions, many of which encode proteins of the immune system and are highly polymorphic [32]. Based on this consideration and our previous findings [16], we reason that natural variations associated with these genes should tend to be neutral and labeling them with a specified feature should be helpful to our classifier. To identify the HLA SAPs, we performed Blast with the corresponding protein sequences against the IMGT/HLA database [32]. Those hit by IMGT/HLA entries with both an e-value less than or equal to 0.01 and a sequence identity greater than 70% were assigned as HLA proteins, and their SAPs were assigned as HLA SAPs, accordingly.

Disordered region. In addition to the disorder score calculated by VSL2, we also used disordered region information parsed from DisProt [29]. We did a Blast of the protein sequences against the DisProt [29] database and set the e-value to be less than or equal to 0.01 and the sequence identity to be greater than 70%. Based on the blast hits, we transferred the annotation of disordered regions to the query protein and thereby determined whether the SAPs on this protein were located in disordered regions.

Functional sites. Proteins play their biological roles through functional sites, and an alteration in or near a functional site is more likely to disturb the normal function than alterations at other sites. Based on this consideration, adopting attributes to represent these effects will likely be helpful in solving the SAP classification problem [16], [33]. Similarly to previous methods, we defined these attributes using the sequential distance between SAP and the nearest functional sites (if greater than 50, set 50 as the upper bound). The functional sites used here were taken directly from Swiss-Prot annotations with the feature table key of ACT_SITE, BINDING, CARBOHYD, LIPID, METAL, MOD_RES, CROSSLNK and DISULFID. We also used TRANSMEM annotation, where the attribute was assigned as either 1 or 0 to indicate whether the SAP was in a trans-membrane region or not.

GRANTHAM score. Each element in the GRANTHAM matrix shows the differences of physicochemical properties between amino acids [34]. Using these values, we defined an attribute for each SAP that reflected the physicochemical difference between the original and changed residue.

#### Feature space of SAP

The microenvironment of a SAP consisted of 8 amino acids: 4 neighboring amino acids on each side. Including the original and changed amino acids of the SAP, a total of 10 amino acids were encoded. Hence, each SAP was programmed to have 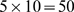 AAFactors, 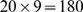 PSSM conservation scores, 1 protein betweenness, 220 KEGG enrichment scores, 9 disorder scores and 12 other structural features; this resulted in a total of 472 features.

### mRMR method

The Maximum Relevance, Minimum Redundancy method [21] was originally developed by Peng et al. The mRMR program used in this paper was downloaded from the website http://penglab.janelia.org/proj/mRMR. It ranks each feature according to both its relevance to the target classification variable and the redundancy between the features. A “good” feature is characterized by maximum relevance with the target variable and minimum redundancy within the features. Both relevance and redundancy are defined by mutual information (MI), which estimates how much one vector is related to another. MI is defined as follows:
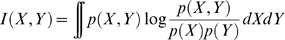
(2)where 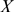 and 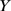 are two vectors, 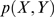 is the joint probabilistic density, and 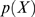 and 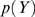 are the marginal probabilistic densities.

Let 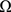 denote the whole vector set. The already selected vector set with 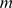 vectors is denoted by 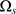, and the to-be-selected vector set with 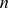 vectors is denoted by 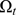. The relevance 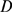 of a feature 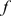 in 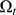 with a classification variable 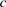 can be computed by equation (3):
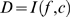
(3)The redundancy 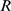 of a feature 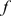 in 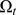 with all the features in 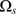 can be computed by equation (4):
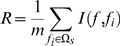
(4)To maximize relevance and minimize redundancy, mRMR function is obtained by integrating equation (3) and equation (4):
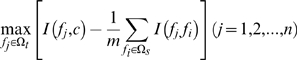
(5)For a feature pool containing 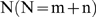 features, feature evaluation will be executed in 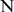 rounds. After the pre-evaluation procedure, a feature set 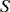 will be provided:
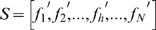
(6)In the feature set 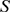, the feature index h denotes at which round the feature is selected. Evaluations for features are also reflected by these indices. For example, 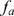 is believed to be better than 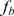 if 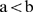 because the better the feature satisfies equation (5) the earlier it will be added to 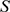.

### Nearest Neighbor Algorithm

In our work, the Nearest Neighbor Algorithm was used to classify each SAP as either neutral or disease-associated. Its basic idea is to make a prediction based on the calculation of similarity between the test samples and the training samples. The distance between two vectors 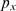 and 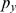 in the study is defined as [35], [36]:
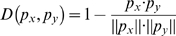
(7)where 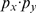 is the inner product of 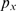 and 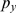, and 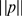 is the module of vector 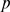. A smaller value of 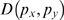 means increased similarity between 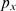 and 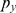.

In NNA, a vector 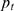 will be designated as having the same class as its nearest neighbor 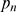, i.e. 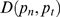 is the smallest distance among all the other distances.
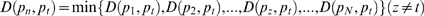
(8)where 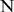 represents the number of training samples.

### Jackknife Cross-Validation Method

The Jackknife cross-validation, also called Leave-One-Out Cross-Validation (LOOCV) [35], [36], [37] is one of the most effective and objective ways to evaluate statistical predictions. In the Jackknife cross-validation Method, each sample in the dataset is knocked out in turn and tested by the predictor, which is trained by the other samples in the data set. During this process, each sample is involved in training 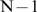 times and is tested exactly once. To evaluate the performance of the predictor, the accuracy rates for the positive samples, negative samples and the overall samples can be calculated as:
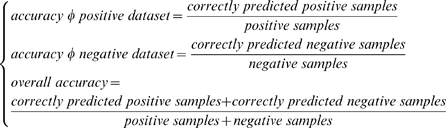
(9)


### Incremental Feature Selection (IFS)

After the mRMR step, we obtained a feature list in their order of selection. However, we do not know how many features in the list should be chosen. In our study, Incremental Feature Selection (IFS) [35], [36] was used to determine the optimal number of features.

We constructed 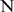 feature subsets of the feature list S provided by the mRMR feature list defined in eq. (6) by adding an additional feature to the candidate feature subset, starting from an initial subset containing only the first feature 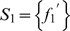. The i-th feature subset is defined as:
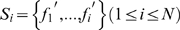
(10)by adding feature 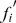 to the previous subset 
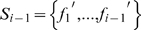


For each feature subset 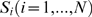, the Jackknife cross-validation method is used to obtain the accuracy of prediction. The results were plotted to produce an IFS curve with index 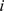 as its x-axis and the overall accuracy as its y-axis. The feature set, say 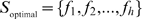, would be considered as the optimal one if the IFS curve has a peak at 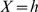.

### Deleterious/tolerated SAP predicted by SIFT

SIFT [38] version 4.0 was downloaded from http://sift.jcvi.org/www/sift4.0.tar. The protein sequences database was downloaded from UniProtKB/TrEMBL Release 40.12; NCBI BLAST version 2.2.22 was used as a search engine. Lists of amino acid substitutions to be predicted were generated and the median conservation was set as 3.00.

## Results

### mRMR result

The first step of feature selection is to produce an mRMR feature list. Because our data is continuous, we set the parameter 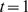 to categorize each feature in our data into one of three possible states according to the equation 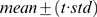: the ones with a value smaller than 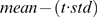, the ones with a value between 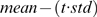 and 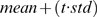, and the ones with a value larger than 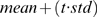. In these formulas, mean is the mean value of the features in all samples and 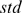 is the standard deviation. All 472 features were ranked according to their importance for prediction by mRMR.

### IFS results

**Figure 1:**
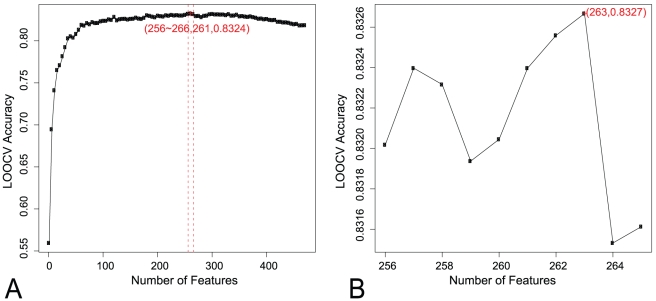
The curve of IFS. (A) The IFS curve with a step width of 5. The highest accuracy was achieved with 261 features, which suggest the optimal feature set should have more than 256 and less than 266 features; (B) The IFS curve between index 256 and 265. Refine the accuracy around *S_261_*, by calculating accuracies using feature sets *S_256_*, *S_257_… S_265_*. The highest accuracy of IFS was 83.27% using 263 features. These 263 features formed the optimal feature set.

As was mentioned in the above section, each SAP was represented by 472 features. A NNA model was built 472 times for the IFS procedure by adding features one by one to the model from the list of 472 mRMR features. **Figure 1** shows the results of IFS. To improve the efficiency of the computation, IFS was executed by alterable steps to search for the highest accuracy as follows:

1. Calculate the accuracy with feature set 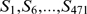 using 5 features as the step.
2. Find the index of the feature set with which the maximum accuracy was achieved, (261 for the data used in this research).
3. Refine the accuracy around 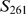, by calculating accuracies using feature sets 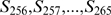.

The highest accuracy of IFS was 83.27% using 263 features. The accuracy of polymorphism SAP and disease SAP classification using these optimized 263 features were 85.26% and 80.73%, respectively. The detailed information of the IFS procedure and the optimized 263 features of IFS are listed in **Table S1** and **Table S2**.

### Independent testing of our method

Human polymorphisms and disease mutations in Release 57.4 on 16-Jun-2009 were used for Jackknife cross-validation. The newly added SAPs in release 57.13 after release 57.4 were used as independent test dataset. The independent test dataset was composed of 1,905 polymorphism SAPs and 766 disease SAPs. The prediction accuracy of the independent test was 80.0%, which was slightly lower than the accuracy of the Jackknife cross-validation on training set, which was 83.27%.

## Discussion

### Comparison with SIFT

To compare our method with SIFT, we analyzed the same data used in our predictor with SIFT. Some SAPs couldn't be predicted using SIFT due to limited diversity among their protein sequences. Among the remaining SAPs, each one was identified as deleterious (“Disease”) or tolerated (“Polymorphism”). The prediction accuracy of SIFT was 71.05%, which is lower than our method.

SIFT (‘Sorting Tolerant from Intolerant’) is based on the principles of protein evolution. Generally speaking, a highly conserved position should be intolerant to most substitutions, whereas a poorly conserved position can tolerate more substitutions [39]. From a query protein sequence, SIFT compiles a dataset of functionally related protein sequences by searching a protein database using the PSI-BLAST algorithm. Then, the sequences that are homologous with the query sequence are used to build an alignment. In this step, SIFT scans each position in the alignment and calculates the probabilities for all of the 20 possible amino acids at that position. These probabilities are normalized by the probability of the most frequent amino acid and are recorded in a scaled probability matrix. SIFT predicts how a substitution affects protein function, based on the scaled probability, by comparing the SIFT score to the threshold value given by user. It was previously reported that, when applied to a dataset of mutations found in individuals affected with a disease, SIFT correctly predicted that 69% of the substitutions associated with the disease affected protein function [40]. The reported prediction accuracy is close to the prediction accuracy of SIFT in dataset of this study.

Unlike SIFT, our methods used more features, including the AAFactors, similarity to HLA families, disorder attributes, distance between SAP and functional sites, betweenness and the KEGG enrichment scores of the protein neighbors. These features incorporated both amino acid- and protein-level information. In particular, betweenness and the KEGG enrichment scores were network level features. The results suggest that it is better to uncover the complexity of diseases by integrating network-centric methodology with the traditional sequence-based methodology.

### Feature analysis

**Figure 2:**
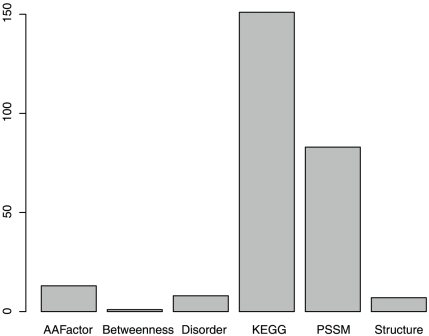
The number of each type of features in the optimal feature set. The feature with the biggest contribution is KEGG enrichment scores, one kind of the network features. Another kind of the network features, betweenness, was also important. This suggests that if a protein does not interact with biologically important proteins, then its mutation may not cause severe damage.

**Figure 3:**
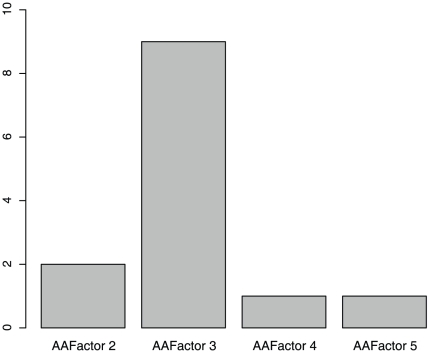
The number of each type of AAFactor features in the optimal feature set. Factor 3 is the most important one and it relates to molecular size or volume with high factor coefficients for bulkiness, residue volume, average volume of a buried residue, side chain volume, and molecular weight.

Some features can improve the prediction accuracy when they are added, while others cannot. **Figure 2** shows the number of each type of feature in the optimized 263-feature set. Since the prediction accuracy already achieved 80.29% with 36 features (see **Table S1**), we also plotted the number of each type of feature in these top 36 features ranked by mRMR in **Figure S1**. As we can see from both **Figure 2** and **Figure S1**, the feature with the biggest contribution is KEGG enrichment scores, one kind of the network features. To more objectively evaluate the importance of KEGG enrichment scores, we did hypergeometric test on the optimal feature set and found the 263 selected features were significantly overrepresented onto KEGG enrichment scores with p value of 9.03×10^−8^. Another kind of the network features, betweenness, was also important. This suggests that if a protein does not interact with biologically important proteins, then its mutation may not cause severe damage. The second most important feature is the PSSM conservation score, which is similar to the basis of SIFT. Conservation is one of the most important concepts in biology. If an amino acid in a particular position of a particular protein is conserved, then it may mean that this amino acid is located in an important or functional region of the protein and that its mutation may cause a significant change in the protein's shape and function. The third most relevant feature is the transformed scores of the amino acid index (“AAFactor”). **Figure 3** shows the frequency of each type of AAFactor features in the optimized 263-feature set. It appears that factor 3 is the most important one. Factor 3 relates to molecular size or volume with high factor coefficients for bulkiness, residue volume, average volume of a buried residue, side chain volume, and molecular weight [31].

The most important single feature is the enrichment scores of KEGG pathway is the hsa04350 TGF-beta signaling pathway. The importance rank of each feature can be found in **Table S2**. Transforming growth factor-beta proteins (TGF-beta proteins) are key players in a large variety of physiological and disease processes. The TGF-beta signaling pathway is related to many cellular processes in both the adult organism and the developing embryo including cell growth, cell differentiation, apoptosis, cellular homeostasis and other cellular functions. If a protein can interact with some proteins in TGF-beta signaling pathway, its mutation has the potential to cause serious damage to the system. The second most important single feature is the disorder score of the site, two amino acids ahead of the SAP. Disordered regions in proteins lack fixed three-dimensional structures under physiological conditions, and they play important roles in regulation, signaling and control, which can involve high-specificity, low-affinity interactions and binding of multiple proteins [29]. Amino acid substitutions that happened in these regions would most likely disturb their normal functions and thus cause a disease phenotype. The third most important single feature is the PSSM conservation score of the SAP site, which is expected. The fourth is the GRANTHAM score. The GRANTHAM matrix shows the differences of physicochemical properties between amino acids [34]. Intuitively, the larger the difference, the more likely the SAP would destroy the function of the protein. We compared the GRANTHAM scores of SAPs annotated with disease to those annotated with polymorphism and found the former ones were greater than the latter, on average. This confirmed our intuition and showed their contribution to our ability to discriminate disease SAPs from polymorphism ones. Betweenness was 20^th^ important as single feature. Betweenness measures the information flow through networks; a high betweenness indicates multiple paths between nodes, and a low betweenness indicates few paths. In a biological network, betweenness measures the ways in which signals can pass through the interaction network.

In this study, careful feature selection and analysis was performed to choose an optimal feature set and to analyze what kind of features are important for detection of deleterious SNPs. Network features were found to be most important for accurate prediction and can significantly improve the prediction performance. Our results suggest that the protein interaction context could provide important clues to help better illustrate SAP's functional association.

## Supporting Information

Table S1 The IFS table.
(0.03 MB XLS)

Table S2 The optimized 263 features.
(0.05 MB XLS)

Figure S1 The number of each type of feature in the top 36 features. With these 36 features, the prediction accuracy achieved 80.29%.
(0.00 MB PDF)
