# Supplementary figures and images for: Prediction of Deleterious Non-Synonymous SNPs Based on Protein Interaction Network and Hybrid Properties
Source: PLoS One. 2010 Jul 30;5(7):e11900. doi: 10.1371/journal.pone.0011900 (PMC2912763; doi:10.1371/journal.pone.0011900)

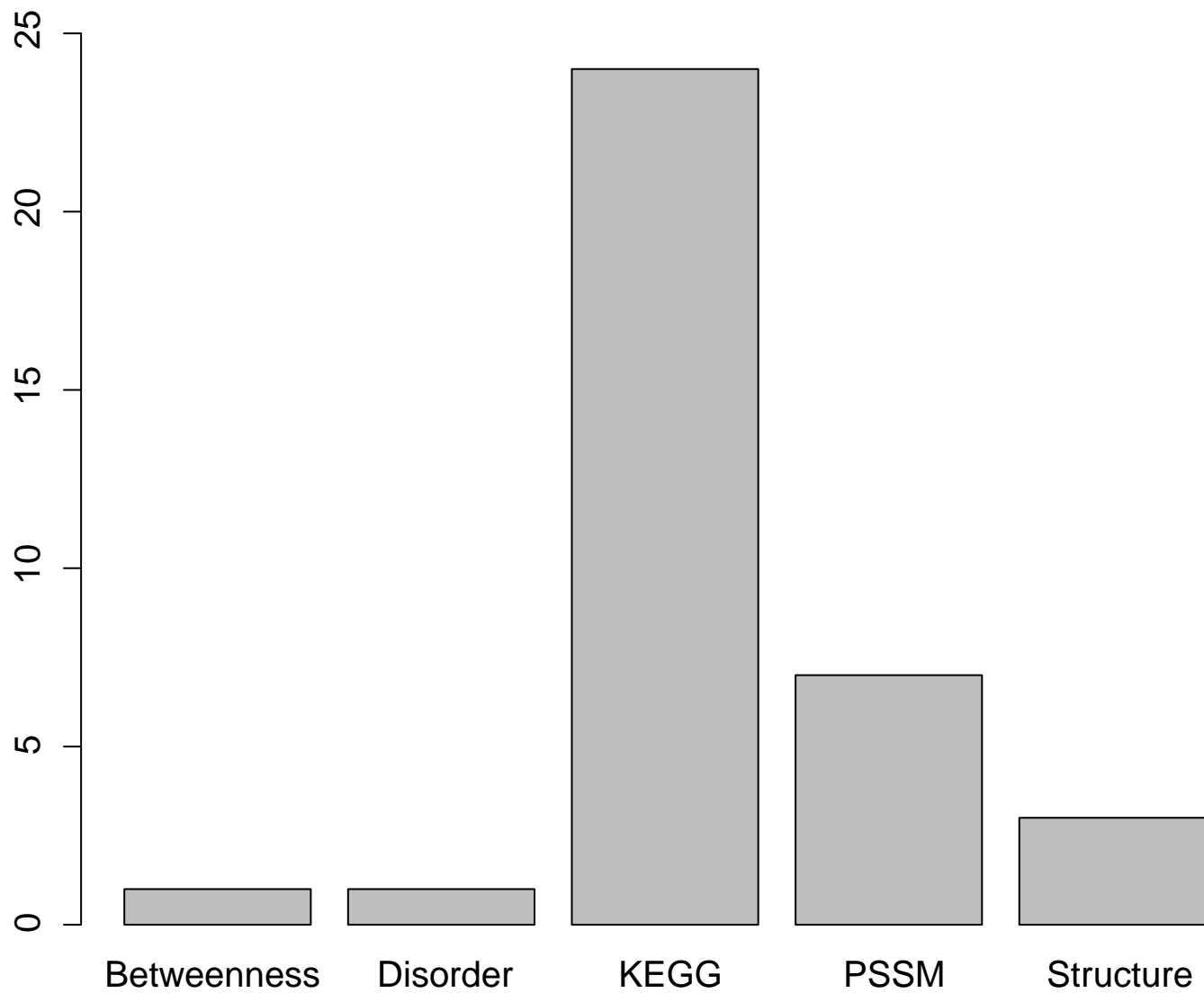

Supplement: Figure S1 — The number of each type of feature in the top 36 features. With these 36 features, the prediction accuracy achieved 80.29%. (0.00 MB PDF) [file pone.0011900.s003.pdf]
